# Obtaining transition rates from single-channel data without initial parameter seeding

**DOI:** 10.1080/19336950.2020.1732004

**Published:** 2020-02-28

**Authors:** Michael Voldsgaard Clausen

**Affiliations:** Aarhus Institute of Advanced Studies, Aarhus University, Aarhus, Denmark

**Keywords:** optimization algorithms, single-channel analysis, root finding, kinetic models

## Abstract

**Background and Purpose:** Ion-channels are membrane proteins that can adopt several distinct structural conformations. Some of the conformations are open and allow the passage of ions through the membrane; others are closed and hinder ion flow. Patch-clamp recordings of single ion-channels show if a channel is open or closed, but does not immediately reveal the underlying mechanism, which typically includes several open and closed conformations.

With kinetic analysis of single-channel data, sequences of observed open and closed times are fitted to proposed schemes to deduct the underlying kinetics of the ion-channel. Current programs to perform kinetic analysis uses initial parameter guessing. Here an alternative approach that uses a global fitting procedure and no initial parameter seeding is developed and tested.

**Methods:** Different fitting algorithms that use variations and combinations of Simplex-optimization, Genetic Algorithm and Particle Swarm are tested against simulated data with brief events removed as in real resolution limited data.

**Results:** A two-step fitting algorithm that uses Particle Swarm optimization to find initial parameters and then a modified Simplex approach to fine-adjust the initial parameters successfully find the correct rates used for data simulation.

**Conclusions:** SCAIM (Single Channel Analysis in MATLAB) facilitate the deduction of kinetic schemes underlying single-channel data.

## Introduction

Ion-channels are membrane proteins that alter between two functional states: open/conducting and closed/non-conducting. Factors that influence a channel’s open-probability, e.g. membrane-voltage, temperature, and various ligands, as well as selectivity to different ions, vary between distinct classes of ion-channels. As a group, ion-channels determine the flow of, e.g. K^+^, Na^+^, Cl^−^ and Ca^2+^ through cellular membranes, and hence play essential roles in ion-homeostasis, excitability, and signaling. Understanding the mechanisms that govern ion-channels movements between different states, therefore, represents a crucial challenge in fields as biophysics, pharmacology, and physiology.

**Figure 1.**
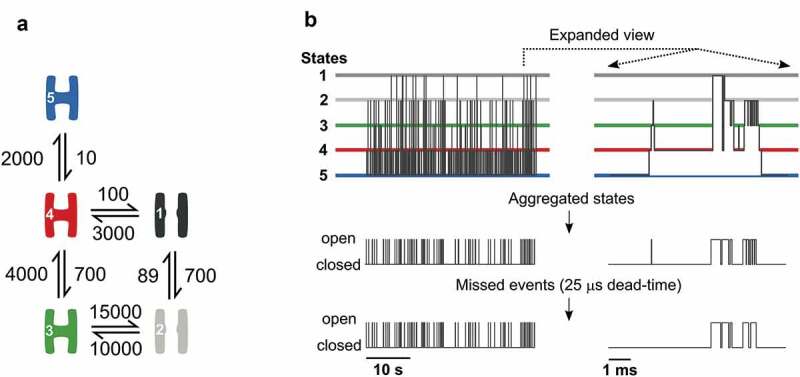
Information loss from kinetic scheme to processed data.a) Kinetic scheme showing the connection framework of a five-state ion-channel with two open (grey and labeled 1 and 2) and three closed conformations (colored and labeled 3, 4, and 5), and transition rates are given in s^-1^. b) The upper panel illustrates how the channel in A moves between the five states as time progresses. The middle panel demonstrates loss of information as only transitions between open and closed states are observed. The bottom panel shows the observed data after the loss of brief events to limited time-resolution.

Patch-clamp techniques allow recordings of single ion-channels [1,2] by fixing the membrane potential and simultaneously observing channel openings as pico-ampere deflections from baseline currents. The near-instant jumps between open and closed states demonstrate how proteins move between a finite number of distinct structural conformations. Thermal motion causes all parts of a protein to fluctuate at a picosecond timescale; the observed open and closed dwell times (µs and up) hence represent average structures around discrete conformations. The energy barriers that isolate distinct states determine both the time a channel dwell in a particular state, and the frequency with which it transits to other states. This information is embedded in kinetic-schemes that specify channel states, connections, and transition rates (Figure 1a).

Ion-channels transit between several open and closed conformations, but single-channel recordings only shows if a channel is open or closed (Figure 1). With this and the assumption that any transition between distinct states depend only on the identity of the current state, ion-channels are well described by aggregated Markov models [3]. An aggregated Markov model is a particular case of the hidden Markov model where the output probabilities are fixed for each state. A closed state has probability 1 of having zero conductance, whereas an open state has probability 0 of having zero conductance [4,5].

While temporal resolution for single-channel recordings is uniquely good among single-molecule techniques, it is still limited to around 10 µs at best [6]; often, it is much worse. A consequence of limited time resolution is a loss of information; some events are too brief for precise detection. Single-channel dwell times are exponentially distributed, so, e.g. with a time resolution of 10 µs, almost a fifth of events with a mean distribution of 50 µs are missed.

Figure 1 illustrates the gradual loss of information, from the ideal ion-channel that transits between five distinct structural conformations, to the observed single-channel recording with missed brief events. Our goal is to backtrack and infer the underlying kinetic scheme from recorded, resolution limited, single-channel data. Currently, two programs that allow this. HJCFIT is freely available and well-tested [7] but requires data for analysis to be in a specific file format, which becomes a limitation for some users. There is no obvious route from data acquired with, e.g. Axon^TM^ Clampex^TM^ to analysis with HJCFIT. The second program MIL is part of the QuB software, which is at this time of writing, no longer freely available. The MIL approach uses an approximate method for missed event correction [8], while HJCFIT uses an exact correction [9] and an asymptotic form [10]. Furthermore, both programs require an initial guess of the transition rates.

The SCAIM (Single Channel Analysis in MATLAB) package presented here include scripts for (i) deduction of kinetic schemes from idealized single-channel data using a simple file format and without initial guessing (ii) a simple threshold-crossing method for idealization of data recorded with Axon^TM^ Clampex^TM^ (iii) a script that impose a fixed dead-time on idealized data (iv) a script for simulation of single-channel data.

## Methods

### Direct fitting of dwell times

The script SCAIM_fit takes as input a list of open and closed dwell times, a proposed scheme that describes how individual states are connected, and statements about parameters that are restricted, fixed or set by kinetic reversibility (see appendix 1). Before the script initiates, a dead-time is imposed on the list of dwell times, so events briefer than the given dead-time gets concatenated with flanking events. The script SCAIM_imposeDT does this.

Several ion-channels comprise closed conformations that become available after, e.g. activation by voltage (inactivated states [11]) or at high agonist concentrations (desensitized states [12]). The mechanisms of inactivation [11] and desensitization [13] may be complex and include several distinct states. Only single-channel data obtained under steady-state conditions are suitable for fitting, and all states with zero conductance belong to the group of closed states regardless if they are inactivated or desensitized.

For direct fitting of a list of dwell times, the global optimization algorithm suggests transition rates for the proposed kinetic scheme, and the direct fitting algorithm evaluates how well the proposed kinetics fit with the observed sequence of dwell times. The procedures for direct fitting of dwell times to a proposed mechanism [14] and correction for missed events [9,10] are given in the cited papers; here, a brief overview is provided.

The finite time-resolution of single-channel recordings cause short events to become inaccurate or even missed. By imposing a dead-time (τ), so that all events briefer than τ gets concatenated with flanking events, the value of τ comes to define the resolution of the analyzed data. The dead-time is set so that all events longer than τ are detected and measured correctly.

The underlying system is modeled by a continuous-time finite-state Markov process, *S(t)*, where *S(t)* =* i* designates that the process is in state *i* at time *t* [15]. The state-space *I* defines the possible states of the system, and each state in *I* is either open (set A) or closed (set F). Transitions between states are encoded and parameterized by the generator matrix Q, a square array with entries in *i*th row and *j*th column. Entries (q_ij_) of the Q matrix, where *i* ≠ *j*, give rate constants in units of reciprocal time for the transition from state *i* to state *j*. The diagonal, where *i* = *j*, are chosen, so the sum of all values in the corresponding rows equals zero. The values of q_ii_ hence become negative, and -q_ii_ comes to represent the total rate at which the channel leaves state *i*. The Q matrix is partitioned for the two conductance levels so that partitions Q_AA_ and Q_AF_ represents rates between open states and rates from open to closed states, respectively. Q_FF_ and Q_FA_ analogously represent transitions between closed and from closed to open states.

In the ideal case, with no missed events [15], observed dwells in open or closed conformations still represent aggregates of the underlying state transitions. The probability density for a process that begins in A, and remain within the set of open states (iϵA), for a sojourn *t*, and then instantaneously transits to a closed state jϵF is given by the elements of the matrix G_AF_(*t*) [15]:

(1) $$G_{AF}\left(t\right)=\exp\left(Q_{AA}t\right)Q_{AF}$$

An expression for G_AF_(*t*) in the realistic case, where all events briefer than τ are missed, comes to depend on the evaluation of a matrix, ^A^R(*u*), with elements *i* and *j* that are part of A. It gives the probability of an opening that begins in state *i*, has not finished after time *u* and is currently in state *j*. The transition density, when considering events briefer than τ as missed, become:

(2) $${\;}^{e}G_{AF}\left(t\right)={\;}^{A}R\left(t-\tau \right)Q_{AF}\exp\left(Q_{FF}\tau \right),\;\;\;t\geq \;\tau$$

The assessment of ^A^R(*u*) follows two separate strategies, the exact evaluation for *t* ≤ 3τ [9], and, as the exact approach is unfit for higher values of t, the asymptotic approximation for *t* > 3τ [10].

The sequence of dwell times of alternating open (t_0_) and closed (t_c_) times is fitted to a proposed transition matrix Q by optimizing the likelihood of the following expression [14]:

(3) $$\begin{array}{rl} & l=Eq_{A}{\;}^{e}G_{AF}{\left(t_{o1}\right)}^{e}G_{FA}{\left(t_{c1}\right)}^{e}G_{AF}{\left(t_{o2}\right)}^{e}G_{FA}\left(t_{c2}\right)\\ & \;\;\;\;\;\;\;\;\;\;{\;}^{e}G_{AF}\left(t_{o3}\right)\ldots u_{F} \end{array}$$

where ^e^G_FA_(t) is evaluated as ^e^G_AF_(t) with all A’s and F’s swapped, u_F_ is a vector of ones, and Eq_A_ is the equilibrium vector [14] which specify the probability that an opening start in each of the open states.

The asymptotic solution depends on numerically localizing a number of roots that correspond to the number of states in the proposed mechanism [16], given that it obeys the principle of microscopic reversibility [15]. The asymptotic behavior of ^A^R(*u*) depends on values of *s* that render singular the matrix W(s) defined as:

(4) $$W\left(s\right)=sI-H\left(s\right)$$

where

(5) $$\begin{array}{rl} & H\left(s\right)=Q_{AA}+Q_{AF}(sI-Q_{FF}{)}^{-1}\\ & \;\;\;\;\;\;\;\;\;\;\;\;\;\;\;\;\;\;\left(I-\exp\left(-\left(sI-Q_{FF}\right)\tau \right)\right)Q_{FA} \end{array}$$

These values of *s* are found as roots of the determinantal equation

(6) $$\det\left[W\left(s\right)\right]=0$$

Lower and upper bounds are determined in a two-step process, where initial estimates are found as minimum and maximum eigenvalues of *H(s)* with *s* set to 1. The initial estimates are then expanded in both ends until they fulfill the following criteria: the sign of det[W(s)] evaluated at the upper and lower bounds are equal with an even number of roots. The signs of the derivatives of det[W(s)] evaluated at the bounds are, on the other hand, opposite with an even number of roots. The reverse applies to an odd number of roots.

Because the global search for an optimal Q matrix assesses a wide range of parameters, the root-finding algorithm must operate with both high speed and fidelity. Here three approaches for numerical root finding are compared:

Method 1 begins with a simple bisection approach that locates brackets that capture single roots. Next, the built-in MATLAB function fzero is used to locate the exact root within each set of brackets.

Method 2 is a Newton-Raphson approach with start points systematically chosen between the initial borders.

Method 3 is a custom modification of the bisection algorithm that evaluates the distance from zero, sign, and direction of det[W(s)] at bracket points. Bracket points are continuously sorted based on these parameters, and the most promising kept while the rest are discarded.

### Parameter optimization algorithms

The function that fits a kinetic scheme with associated parameters to a list of dwell times gives as output a likelihood that quantifies the goodness of the fit. For optimization, the sign of the likelihood is inverted, so the search algorithms seek parameters that minimize the value of the dwell-time fitting function, from here referred to as the fitness function.

To optimize *n* parameters when an initial guess is provided, the script uses the Nelder-Mead Simplex (NM) method [17], which continually compares function values of *n* + 1 vertices of a general simplex. By alternating between rearranging parameter estimates based on their function value and modifying the lowest ranking estimates using information from higher-ranking, the simplex converges toward a local minimum. Here the original approach is modified to explore the parameter space around the initial guess better. Once the simplex comes close to a minimum, the parameters are disturbed, and the optimization restarted. The degree of disturbance, restart threshold, and number of restarts are adjustable parameters. The final run continues until the function value no longer improves, or the maximum of iterations reached.

Two approaches from the MATLAB global optimization toolbox are evaluated: Genetic Algorithm [18] (GA) and Particle Swarm [19] (PA). The GA modifies an initial population of solutions through the production of offspring and mutants that become the population in the following generation (iteration). Based on output from the fitting function, the best individuals (20%) from a generation produce children of the next generation by parameter crossing. Each generation preserves an elite (5%), the individuals with the best function values. The remaining individuals have their parameters mutated randomly to produce mutants of the new generation.

PS treats an initial population of solutions as individual particles with a position and a direction in the parameter-space. The position of individual particles correspond to its current parameter values, and the position is hence associated with a fitness function value. Each particle recall its best prior position and know the overall best position visited by any particle of the swarm. At each iteration, the position and direction of individual particles are updated: a new position is based on prior direction and position, and a new direction is set based on prior direction, best prior position and the overall best position of the entire swarm.

### Simulation of data

Here two simulated datasets are evaluated. The SCAIM_sim script constructs a Q matrix based on input information (appendix). The MATLAB random number generator provides random uniformly distributed numbers, *r*, between 0 and 1. These random numbers determine dwell times at specific states as (−1/q_ii_)ln(*r*), and, if the state is connected to more than one other state, also with a probability determined by transition rates from the state, which next state is visited.

For the five-state model, 322,466 state transitions were simulated and then aggregated into 29,918 transitions between open and closed states. Finally, a dead-time of 50 µs was imposed leaving 15,902 events used throughout this paper.

The seven-state model data is based on 1,028,387 state transitions, aggregated into 42,449 dwell times, and finally reduced to 19,461 events after a 25 µs dead-time was imposed.

## Results

### Finding roots

**Figure 2.**
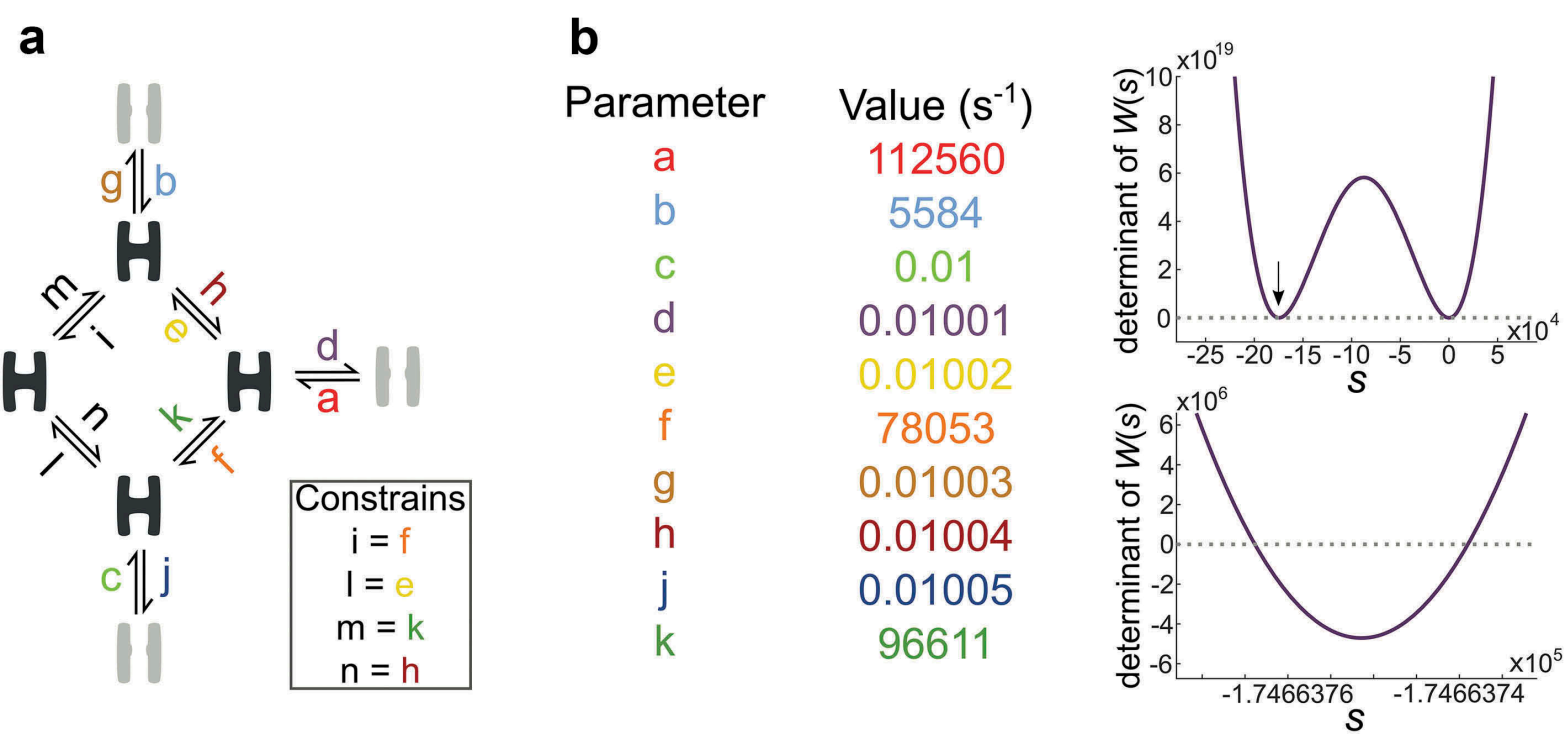
Numerical root finding. The challenge of numerically locating multiple roots is a balance between search resolution and computation time.a) Seven-state kinetic scheme with three open (grey) and four closed conformations (black). The identity of the 14 transition rates is noted as color-coded small letters from a to n with constrained parameters in black. b) The left-hand table gives example parameters for input in the model shown in A. With these parameters; the four roots are located as demonstrated in the right-hand plots. The lower plot is an expansion of the upper plot at the region indicated by the arrow.

For each evaluated model, the asymptotic approach requires the determination of two sets of roots, one with roots corresponding to the number of open states, and one for the closed states. Effective optimization hence requires a root-finding algorithm that locates the roots fast and comprehensively. To compare the capabilities of bisection, Newton-Raphson, and modified-bisection, the root-finding algorithms are applied as PS evaluates 100 particles over 25 iterations. The three procedures were evaluated based on the time they used to locate the four roots associated with the closed states of the scheme given in Figure 2(a). With a two seconds failure-threshold, that abort and register inefficient root-finding attempts, the mean root-search times, with standard deviations in parenthesizes, (for times < 2 s) were 0.011 s (0.073 s) for bisection, 0.268 s (0.0386 s) for Newton-Raphson and 0.098 s (0.042 s) for modified-bisection. During these 2500 parameter evaluations, the 2 s threshold was crossed eight times by bisection, 639 times by Newton-Raphson, and zero times by modified-bisection.

Figure 2(b) highlights an example of parameters that cause bisection to cross the 2 s threshold. When given the time, bisection does find the correct roots after 35 minutes; modified-bisection locates them in 0.9 seconds. The root-finding algorithm in SCAIM_fit utilizes the superior speed of bisection but takes advantage of the reliability of modified-bisection when root-finding takes longer than a second.

### Finding parameters using a local optimization simplex approach

**Figure 3.**
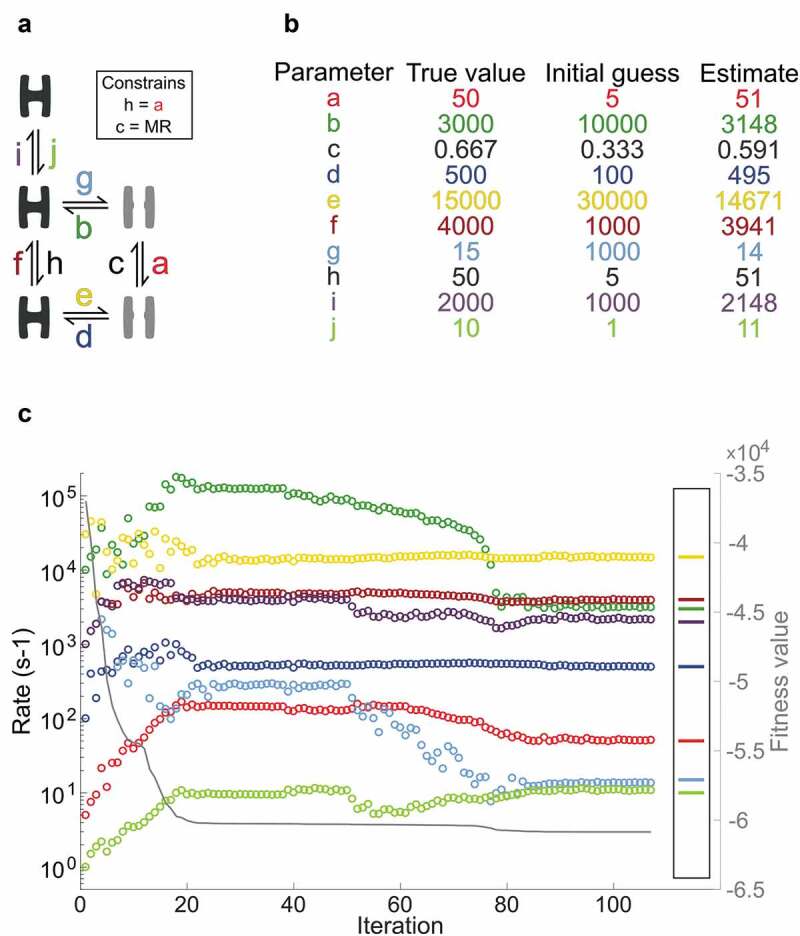
Nelder-Mead optimization with an initial guess. a) Five-state kinetic scheme with two open (grey) and three closed conformations (black) and ten transition rates given as color-coded small letters from *a* to *j* with constrained parameters in black. MR denotes that parameter *c* is set to maintain microscopic reversibility. b) The table shows how NM with a basis in an initial guess estimate parameter values that approach the true values. c) NM improves the parameters given as an initial guess over 510 iterations; of these, those that change the fitness value are included in the plot. The eight parameters are color-coded as in a and b, and the right-hand insert shows the true values. The solid grey line follows the right-hand Y-axis and illustrates how the fitness value improves.

Before trying a global parameter search, the basic functionality of SCAIM_fit was evaluated on the five-state model [14,15] with an initial guess and the local optimizer NM (Figure 3). The simulated dwell-time data is described in the Methods section. With parameter *h* fixed to *a, c* set by microscopic reversibility, and the eight remaining parameters seeded as an initial guess, the script approach the true values over 510 iterations (Figure 3c). The plot tracks the development of individual parameters and the concurrent fitness value given as a solid line with values on the right-hand y-axis. After an initial steep improvement in fitness value, the rates remain almost unaltered between iteration 20 and 37, before all parameters are disturbed at iteration 38, and NM restarted. Without these restarts, the algorithm gets stuck in local minima with sub-optimal parameter settings.

### Global parameter optimization using the genetic algorithm and particle swarm

**Figure 4.**
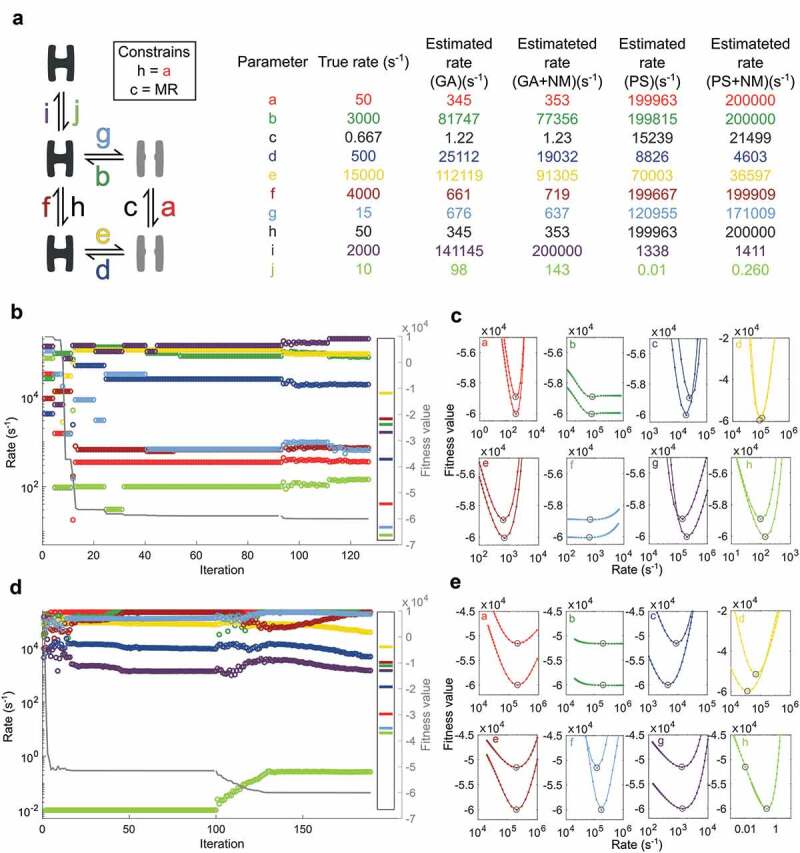
Rate optimization with GA and PS before NMa) Scheme specifying how three closed (black) and two open (gray) states are connected with rates labeled as small letters with color-codes that apply to all figure panels. The box specifies constrained parameters, MR is short for microscopic reversibility. The right-hand table gives rate values for individual parameters. The table shows initial rate estimates by GA and PS, and the final estimates from NM using the best GA or PS estimate as input. b) Progress of GA fitting with the best set of parameters from a population of 200 is plotted over 92 iterations. The remaining iterations are from NM optimization based on the best GA solution. From a 300 iteration NM optimization, only 35 iterations where the fitness value changes are included. The eight parameters are color-coded as in a and b, and the right-hand insert shows the true values. The solid grey line follows the right-hand Y-axis and illustrates how the fitness value improves. c) Each panel illustrates how the function value changes with the given parameter when the remaining rates are fixed. The upper values in each panel are based on the final GA estimate while the lower values are from the final NM estimate. d and e as b and c with PS instead of GA.

Figure 3 shows that NM return rate estimates close to the true values when provided with an initial guess. Figure 4 explores how well two global optimization algorithms, GA and PS, substitute an initial guess. The overall purpose is to let the global optimization run without an initial guess, but for comparative reasons, GA and PS were seeded with the same set of 200 random parameters within the bounds of 0.01 to 200,000 s^−1^.

The step-wise development of GA (Figure 4b) is reminiscent of the elite being preserved from one generation to the next. The smooth development of PS (Figure 4d), on the other hand, stems from the constant movement of all particles, including the best from the former iteration. Both GA and PS converge toward unique local minima that NM further optimizes to new unique minima (Figure 4c,e). The observation that each of these four local minima have parameters that diverge substantially from the true rates, but fitness values that come close, illustrate the importance of correct initial seeding. The best fitness value of the initial set of random parameters is 9901 and the worst 628,890.

With PS optimization, *j* immediately settles at the lowest possible value, and linger at this extreme while the remaining parameters converge toward values that minimize the fitness function when *j* is stuck at 0.01 s^−1^. This tendency was observed during several trials (not shown). The minima plot (Figure 4e) reveals that, by the end of PS optimization, the minimal value of *j* is not optimal; an increased value would improve the fitness function output. NM immediately takes advantage of the possibility missed by PS, and improve all parameters to a new minimum where all parameters are at local minima values. While this immediately suggests a weakness of PS optimization, that particles get absorbed at the boundary conditions, it also demonstrates a promising feature; PS rapidly identifies a parameter with the essential property of keeping the longest closet dwell-time long.

### Optimization with expanding limits

**Figure 5.**
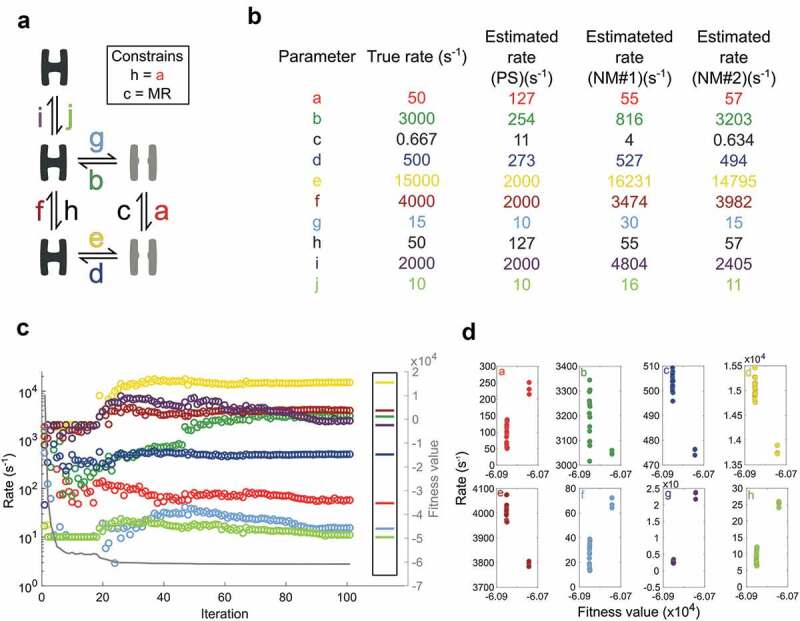
Rate optimization with PS, NM and expanding limitsa) Scheme specifying how three closed (black) and two open (gray) states are connected with rates labeled as small letters with color-codes that apply to all figure panels. The box specifies constrained parameters; MR is short for microscopic reversibility. b) Rate values for individual parameters. The table shows the true rates that simulated data is based on and rates after an initial PS search with limits 10 to 2000 s^-1^. Furthermore, two consecutive rounds of NM optimization improve the best ranking PS estimate, one with limits 1 to 20000 s^-1^ (NM#1) and one with limits 0.1 to 200000 s^-1^ (NM#2). c) Rate estimation begins with 19 rounds of PS optimization; each iteration represents the best estimate of 100 particles. Iteration 20 to 45 covers the first round of NM optimization; the remaining follow the final round of NM. Only iterations where the fitness value change are included in the plot. The right-hand box shows the true values while the right Y-axis and solid grey line track the progressing fitness value. d) Plots for each parameter showing the results of 20 optimization trials.

In Figure 5, PS and NM combines with a stepwise expansion of the border conditions. With initial lower and upper limits of 10 and 2000 s^−1^ PS identifies a set of parameters (PS in Figure 5b) that serve as a starting point for NM optimization. The borders are expanded by factor 10 in both directions, so the lower and upper limits become 1 and 20,000 s^−1^. The starting parameters detected by PS improves with NM optimization (NM#1 in Figure 5b). The lower and upper limits are further expanded by a factor 10 to 0.1 and 200,000 s^−1^ and optimized by NM (NM#2 in Figure 5b).

The plot in Figure 5(c) demonstrates how the PS algorithm immediately anchor parameter *j* at the lower limit with a substantial improvement in the fitness value. In the following iterations parameters *e, f*, and *i* linger at the upper limit witch correspond with their relatively elevated true rates. With these four parameters sorted as either low or high-end rates, the remaining parameters, with the exception of *b*, find values within a factor three of their true values. With this starting point, two rounds of NM optimization bring all parameter values close to their true rates.

**Figure 6.**
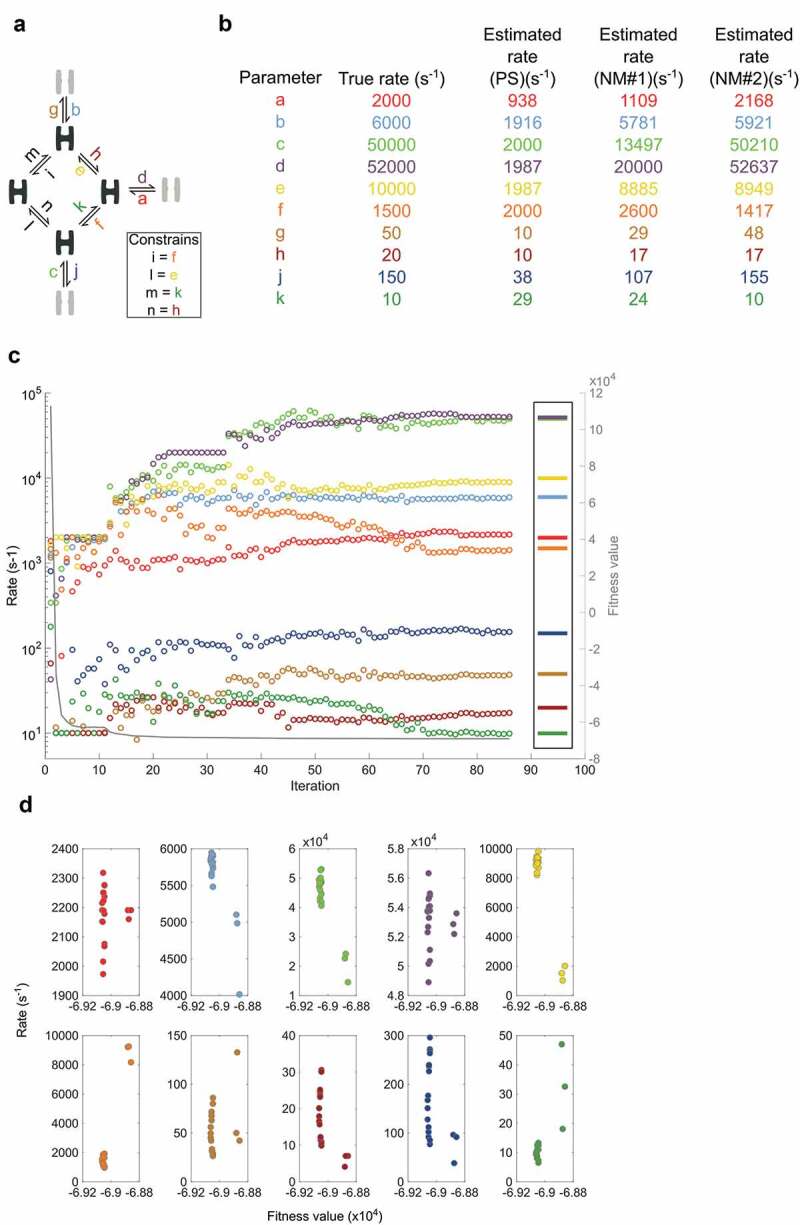
Seven-state model optimization with PS, NM and expanding limitsa) Scheme specifying how four closed (black) and three open (gray) states are connected with rates labeled as small letters with color-codes that apply to all figure panels. The box specifies constrained parameters. b) Rate values for individual parameters. The table shows the true rates that data simulation is based on. Third column show rates after an initial PS search with limits 10 to 2000 s^-1^. Furthermore, two consecutive rounds of NM optimization improve the best ranking PS estimate, one with limits 1 to 20000 s^-1^ (NM#1) and one with limits 0.1 to 200000 s^-1^ (NM#2). c) Rate estimation begins with 11 rounds of PS optimization; each iteration represents the best estimate of 100 particles. Iteration 11 to 33 covers the first round of NM optimization; the remaining follow the final round of NM. Only iterations where the fitness value change are included in the plot. The right-hand box shows the true values while the right Y-axis and solid grey line track the progressing fitness value. d) Plots for each parameter showing the results of 20 optimization trials.

Figure 5(d) tests the robustness of the approach. Out of 20 tests, 17 results in fits where all parameters are within a factor three of the true parameters, while three tests deviate more but also settle at poorer fitness values.

Figure 6 tests the approach with expanding limits against a seven-state model based on the nicotinic acetylcholine receptor [7]. As with the five-state model in Figure 5, PS is efficient in finding parameters that belong in the low and high-end of rates (Figure 6b,c). With a step-wise increment of the limits, NM successfully identifies the ten parameters without any initial guessing or fixed values provided.

Figure 6(d) show that of 20 runs, SCAIN_fit correctly identify all parameters in 17 while the three that are less successful also suffer from poorer fitness values.

## Discussion

Kinetic analysis of single-channel recordings is a uniquely well-suited technique to study protein function in detail. Patch-clamp approaches allow the experimenter to directly observe openings and closures of a single channel at a µs time scale for many minutes or even hours. This is unprecedented; no other technique enables the extraction of such rich kinetic information. However, the deduction of the full information from a sequence of dwell times require advanced probabilistic and mathematical tools. While most of the methods used in the SCAIM_fit script build on well-described techniques for correcting for missed events [9,10] and fitting a model to a sequence of dwell times [14], the global parameter search and root-finding approach are novel.

For each parameterized model fitted to the sequence of dwell times, roots to det[W(s)] corresponding to the number of states are required. With a global search for parameters, the root-finding algorithm must deal efficiently with extremely parameterized models, as the example in Figure 2 demonstrate. With bisection, the number of bracket points increases exponentially with search iterations, so if two roots are numerically similar and the root search space wide, the approach becomes ineffective. SCAIM_fit deals with this problem using a modified bisection algorithm that continuously sort and discard lesser promising bracket points. Sorting comes at a price in time, which in most cases, makes simple bisection a better option. However, if bisection is allowed to stall at difficult root problems, computation becomes inefficient.

Here two global search approaches are tested for their ability to replace the user-dependent initial guessing on which the two alternative fitting programs HJCFIT and QUB rely. Figure 4 shows examples of how PS and GA get stuck at local minima with rate estimates far from the true values. The fitness values after optimization with NM, on the other hand, approach the global minimum (Figure 4e vs. Figure 5d). This suggests that the parameter landscape can contain several wrong rate combinations that return promising fitting values and hence highlight the importance of qualified parameter guessing when such are required. Notably, unrealistic parameter estimations will not match, e.g. single-channel dwell time histograms, and are therefore easily dismissed by the alert experimenter.

Figure 5 demonstrates that a stepwise opening of the full parameter space dramatically facilitates the identification of correct rate estimates. When the search space of PS is limited below the full extent of the true parameters, the algorithm successfully determine the trends of several parameters (Figures 5 and 6b). With these trends, established, consecutive rounds of NM optimization with gradually increasing limits bring the PS estimates close to the true values. Figures 5d and 6d demonstrate the robustness of the approach but also show the importance of evaluating several fitting experiments.

## Conclusion

Analysis of single-channel data is a unique approach to gain unprecedented insights too how proteins operate. The SCAIM package described here provides an opportunity for general users to deduce kinetic schemes from single-channel data.
